# The impact of uncertainty on bereaved family’s experiences of care at the end of life: a thematic analysis of free text survey data

**DOI:** 10.1186/s12904-021-00748-9

**Published:** 2021-04-13

**Authors:** Jackie Robinson, Caitlin Pilbeam, Hetty Goodwin, Deborah Raphael, Susan Waterworth, Merryn Gott

**Affiliations:** 1 Faculty of Medical Health Sciences, School of Nursing, University of Auckland, Auckland, New Zealand; 2 Nuffield Department of Primary Care Health Sciences, University of Oxford, Oxford, UK

**Keywords:** Cardiac disease, Palliative care, End of life care, Uncertainty

## Abstract

**Background:**

Inequities in the provision of palliative care for people with cardiac disease have been well documented in the literature. Despite experiencing significant palliative care needs, those with cardiac disease are less likely to be referred to specialist palliative care services and more likely to die in a hospital when compared to those with cancer. The unpredictable trajectory of heart failure has been identified as a key barrier to providing palliative care with many people experiencing a long period of stability with appropriate medical treatment. However, as the disease progresses and cardiac function deteriorates, exacerbations of acute decompensation can lead to what is often perceived to be ‘sudden’ death. The aim of this study is to explore the impact of uncertainty on how death is remembered by bereaved family members of people with heart disease.

**Methods:**

Thematic analysis of free text collected during a postal survey of bereaved family’s experiences of healthcare services in the last 3 months of life using the New Zealand version of the VOICES questionnaire was undertaken. Data was analysed using a three-dimensional conceptual framework of “scientific uncertainty”.

**Results:**

Eight hundred and twenty-seven completed questionnaires were received of which 12.6% (*n* = 105) indicated that they had cared for someone at the end of life with cardiac disease. Experiences of uncertainty were found to have a significant impact upon bereaved family. Four key themes were identified; distrust in healthcare professionals, stories left incomplete, loss, regret and missed opportunity and disempowerment.

**Conclusions:**

This study highlights the ongoing impact on bereaved family when uncertainty is not made explicit in conversations regarding end of life for people with heart disease. Timely and sensitive conversations regarding the uncertainty of when death may occur is an important factor in ensuring that bereaved family are not left with unresolved narratives. Reframing how we think and talk about uncertainty in end of life care is important, as clinicians’ uncertainties may not always reflect or match up with families’ uncertainties. Being explicit about our inability to be certain about the timing of death may thus lead to a more positive and complete experience for bereaved family.

## Background

Inequities in the provision of palliative care for people with cardiac disease have been well documented in the literature [1]. Despite experiencing significant palliative care needs [2], those with cardiac disease are less likely to be referred to specialist palliative care services and more likely to die in a hospital when compared to those with cancer [3]. The unpredictable trajectory of heart failure has been identified as a key barrier to providing palliative care [4]. Many people experience a long period of stability with appropriate medical treatment. As the disease progresses and cardiac function deteriorates, exacerbations of acute decompensation can lead to what is often perceived to be ‘sudden’ death [5].

Palliative care within the context of heart failure is therefore characterized by uncertainty, a topic which has become increasingly discussed in the literature, particularly in relation to communication of prognostication [6]. Indeed, some have argued that the need for certainty is embedded in the way in which medicine is taught and diagnoses are made. This need for certainty has resulted in a level of discomfort in medical culture in acknowledging uncertainty, despite clinicians being aware of its existence in clinical practice [7].

Clinicians adopt various strategies in an attempt to relieve uncertainty for patients and families. For example, a recent study found that clinicians used ‘absolute categorical time estimates’ (hours, days, weeks) in an attempt to relieve prognostic uncertainty for relatives of people with heart failure. At the same time, relatives helped to relieve the burden for clinicians by expressing their knowledge of uncertainty in their requests for information [8].

Evidence has shown that when uncertainty is suppressed or ignored there can be negative outcomes for patients, families and health care professionals. For example, a study by Etkind et.al [6] found that uncertainty can influence the way in which patients experience advanced illness by impacting on the way in which their information needs, preferences and future care priorities are addressed [9]. Furthermore, it has been argued that when health professionals suppress or ignore uncertainty this can impact negatively on healthcare resources and patient and family experiences [7, 10]. However, whilst negative experiences of care at the end of life have been shown to impact on bereavement recovery [11], there is little evidence about how uncertainty impacts family experiences both at the end of life and during bereavement.

The aim of this research is to explore the impact of uncertainty on how death is remembered by the bereaved family members of people with heart disease, in order to better inform end-of-life conversations between clinicians, patients, and relatives.

### Study design

Participants comprised of bereaved family who had cared for someone at the end of life with cardiac disease in one large urban area of New Zealand and who had participated in a self-completed postal survey regarding their experiences of care in the last 3 months of life. Participants completed the Views of Informal Carers Experiences Survey (VOICES) short form [12], previously adapted to the context of Aotearoa New Zealand [13]. The survey consists of questions regarding experiences of services received in the last 3 months of life, and additional space is provided to enable respondents to enter free text comments about any aspect of the care they received. In addition, demographic data regarding the respondent and the deceased were collected. This paper draws on analyses of the free text data collected in response to three questions:

1. What if anything was good about the care?
2. What if anything was bad about the care?
3. Please use the space below if there is anything more you would like to say about the care provided.

A copy of the NZ version of the VOICES questionnaire [13] was posted to bereaved family with a letter of invitation from the health provider and a post-paid return envelope. A follow-up phone call was made by a nurse employed by the service provider 2 weeks after the questionnaire had been posted to offer people opportunities to ask questions about the study. All study protocols were approved by the University of Auckland Human Participants Ethics Committee (UAHPC: 017662).

We initially planned to send invitations 6–12 months post bereavement, however delays were experienced due to: 1) a need for more follow-up phone calls than anticipated; 2) reposting of surveys to second listed next of kin; and 3) the study overlapping major holiday periods in NZ. Invitations were therefore sent 6–21 months post bereavement. Participants were asked to indicate their consent to participate at the start of the survey.

### Data analysis

Descriptive statistics were used to provide an overview of the study population. Analyses were conducted using IBM SPSS Statistics 25. A thematic analysis of qualitative data was undertaken using a process outlined by Braun and Clark [14]. Familiarisation with the data involved reading and re-reading the data as a team to become immersed in and familiar with its content. During this process, ‘uncertainty’ was recognised as an important emergent issue. Following this initial review, a coding frame was developed and applied to all transcripts by JR, codes were then examined to identify broader patterns of meaning or themes. Review of emerging codes and themes were undertaken by the research team as a whole who met regularly as the data analysis process progressed.

### Conceptual framework

**Fig. 1:**
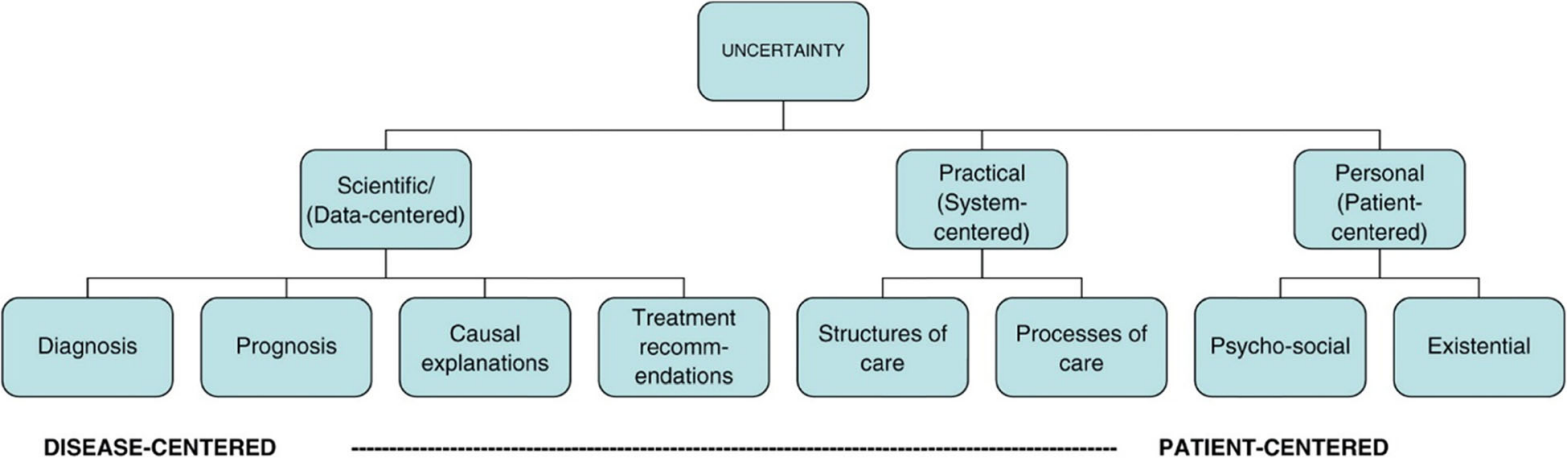
Issues of uncertainty in healthcare [16]

The use of a conceptual framework in qualitative research provides insight into observations and data that might be overlooked or misinterpreted [15]. During analysis, we used a three-dimensional framework described by Han et al. [16], which characterises uncertainty in a healthcare context from different perspectives. The purpose of using this framework was twofold. Firstly, the framework helped to define and identify experiences of uncertainty from within the data. Second, it guided the interpretation of these experiences, helping make sense of how uncertainty impacts on people’s experiences of care at the end of life (see Fig. 1).

## Results

Four thousand seven hundred seventy-eight deaths were recorded during the study period. Of those, 18% (*n* = 860) had incomplete next of kin recorded and 6% (*n* = 235) were returned “not known at this address”, leaving a total of 3683 surveys that could have been completed. Eight hundred and twenty-seven completed questionnaires were returned (response rate calculated 827/3683 = 22.5%); 12.6% (*n* = 105) indicated that they had cared for someone at the end of life with cardiac disease (see Table 1). Open text responses consisted of 4224 words. Some participants provided additional pages to the free text section suggesting that this was seen as a valuable opportunity to share their experiences.

**Table 1:** Participant characteristics (*n* = 105)

Characteristic	Category	Frequency	%
Survey respondents			
Age	40–49 years	8	7.6
	50–59 years	20	19.0
	60–69 years	40	38.1
	70–79 years	26	24.8
	> 80 years	10	9.5
	Missing	1	1.0
Gender	Male	22	21.0
	Female	82	78.1
	Missing	1	1.0
Relationship	Spouse	30	28.6
	Child	55	52.5
	Sibling	7	6.7
	Parent	2	1.9
	Friend	6	5.7
	Other	4	4.7
	Missing	1	1.0
Deceased person’s characteristics			
Age at death	40–49 years	3	2.9
	50–59 years	2	1.9
	60–69 years	6	5.7
	70–79 years	18	17.1
	> 80 years	75	71.4
	Missing	1	1.0
Gender	Male	46	43.8
	Female	58	55.2
	Missing	1	1.0

**Fig. 2:**
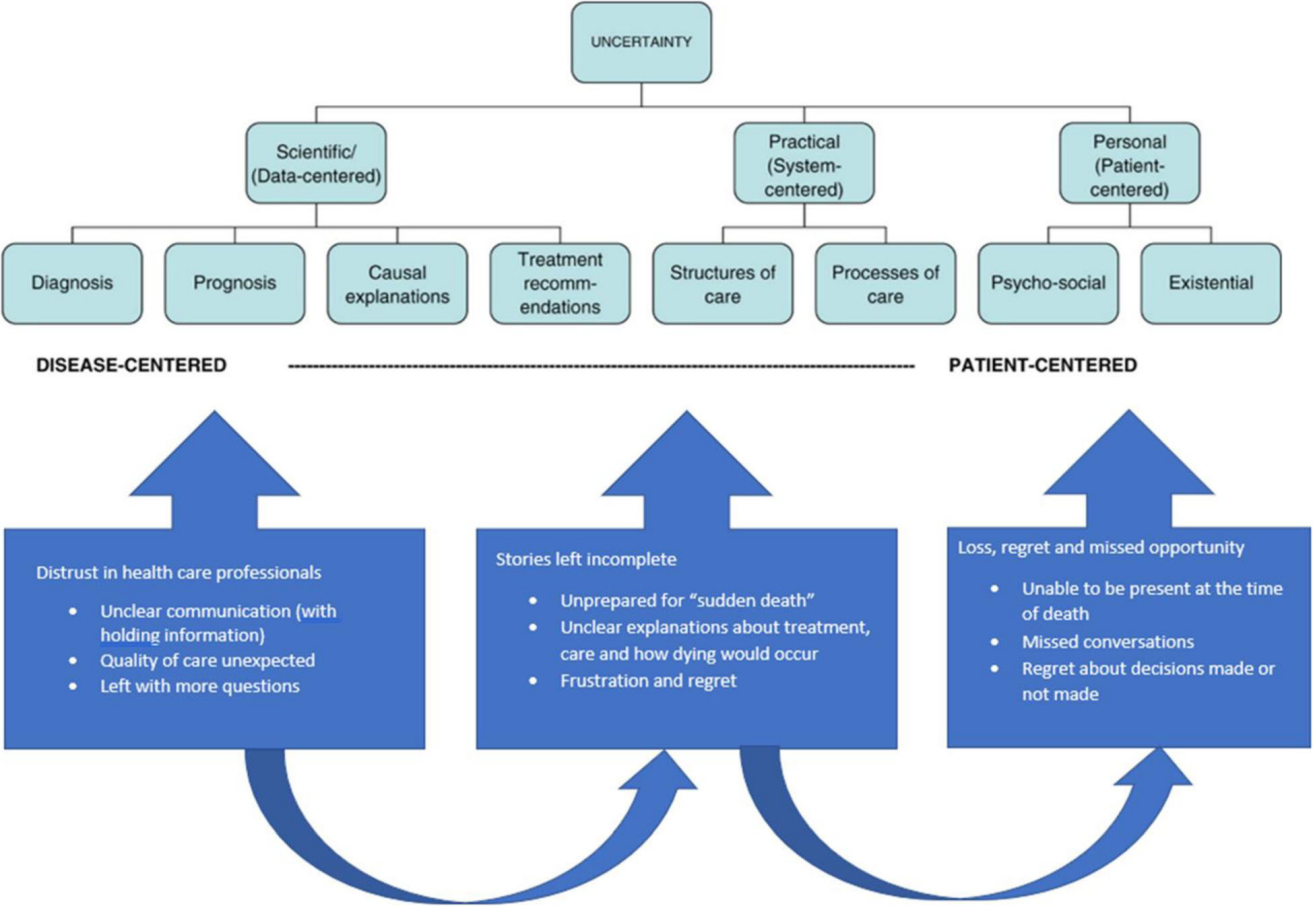
The inter-relation of issues and sources of uncertainty [16] and its impact on bereaved family’s experiences of care at the end of life in cardiac disease

Thematic analysis of the data identified that when uncertainty regarding the prognosis and management of heart disease is not communicated clearly, physical deterioration and death can appear unexpected to family members. This had a significant impact upon family, and four key themes related to this impact were identified and are presented in relation to Hans et.al’s conceptual framework of uncertainty (see Fig. 2) [16]:

1. Distrust in healthcare professionals
2. Stories left incomplete
3. Loss, regret and missed opportunity
4. Disempowerment

### Distrust in healthcare professionals

The three dimensional view of uncertainty in healthcare, described by Han et al. [16], describes “scientific uncertainty” as a source of uncertainty arising from factors associated with diagnosis, prognosis, causation, and treatment. Decision making in this context is characterized by probability, ambiguity, and complexity.

The impact of clinicians not being explicit about prognostic uncertainty, for some participants, led to a sense of distrust about the information being provided. Uncertainty related to the competence of healthcare professionals and the quality of care expected has been described by Han et. Al as “practical uncertainty” [16]. For example, one participant described how she felt that an early discharge from hospital led to her husband’s untimely death at home, and how she had questioned the outcome, given what she had been told about the illness.

00600*: My husband was in NSH and was discharged Sunday afternoon, and three hours later he died at home. I strongly believe that with all the medical information I have read and have had explained to me, my husband may be alive today.*

In addition, family often perceived that healthcare professionals were withholding information when clinical deterioration was occurring, and contributed to family not trusting healthcare professionals’ knowledge. For example, not being “open” about imminent death led one participant to believe that the staff could not be trusted to recognise when someone was imminently dying. The consequence was that family were not present when she died.

*03081: … based on the care of patients, especially those close to death, I think the staff could have been more open about how much time she had left. I think that there are signs when the body is shutting down and staff, having the experience of caring for the elderly could possibly estimate how long patients have left* e.g. *discoloration in feet and hands etc … not specific times as no one knows but estimated. I think her children would have had the opportunity to see her before her passing as they were not living in Auckland.*

When healthcare professionals did not communicate clearly and used language that was not understood to be about dying, family were left feeling as if information was being deliberately withheld. Words such as “final stages” left one participant with even more uncertainty about what to expect.

*04075: We weren’t actually told she was dying, she spent one month only on oxygen. Near the end we were offered palliative care people, they spoke with our Dad who wasn’t accepting that Mum wouldn’t recover. My sister and I were not impressed with them, outward appearance* etc. *When in hospital I requested timeline but very difficult to get information. Dr said she is in final stages of HF, I asked how much time this meant … months, weeks days, no one would answer and give me an idea.*

### Stories left incomplete

A lack of causal explanations is another component of “scientific uncertainty” described by Han et al. [16], the consequences of which left participants in this study with stories left incomplete. Without clear explanations from healthcare professionals, uncertainty regarding treatment, care, and how dying occurred led to unanswered questions for many participants. One participant questioned whether a change in medication may have led to their family member’s death.

*02575: I have a problem with his last admission. Just as the Dr was leaving his room she came back and added a pill. He was discharged not seeing how the pill would affect him and he died about 4 days later. Was this done to end his life!! That’s what I am trying to live with.*

The suddenness of death, for which participants were often not prepared, left some participants questioning whether they had done enough for their family member. One participant described her experience as “bewildering,” and several months after her father’s death expressed that she still felt uncertain about whether she should have asked for more help for him.

*02471: It was a bewildering experience, maybe I could have requested more care but would it have helped him much? I didn’t think so at the time.*

Even when death had occurred, unclear and uncertain information relayed to family left one participant shocked and frustrated, having to work out for herself that her mother had died.

*01585: It was a shock when she died suddenly - and I received the news in a very roundabout way. Called by one nurse to say Mum had a bad night and some difficulty. When I was later called by the Charge Nurse, I had to work out for myself that Mum had died.*

Uncertainty regarding how their family member had died when they were unable to be present at the time of death left one participant with thoughts of her aunt being distressed as she struggled for breath.

*01585: I wish we had been called in order to be with her when she died. I got the impression that she struggled for breath and was in distress for at least 2 h before she died.*

### Loss, regret, and missed opportunity

Ambiguity regarding diagnosis and prognosis is described by Han et al. as another source of “scientific uncertainty,” [16] and for participants in this study, ambiguity in communication with healthcare professionals had a significant impact on participants’ experiences of care at the end of life. When communication regarding prognosis was unclear or occurred ‘too late’, participants experienced loss, regret, and expressed that they felt that there had been missed opportunities.

*00976: I was not notified of impending death and it was 2 h after that I was notified. Very angry about this*.

For another participant, the lack of recognition by staff that the patient was nearing the end of their life meant that the conversation regarding impending death happened very late in illness progression. For some participants, this left them feeling that healthcare professionals withheld knowledge that their family member was dying. The impact of perceived withholding of information meant that one participant was unable to be present when her mother died.

*01571: They did not keep me informed that Mum had deteriorated over the weekend. I think there was a nurse giving better care but my impression of residential care nurses is not positive, they do not appear to have a concept of palliative care. Weekends are particularly poor in care. No one takes responsibility, I would have liked to be there with my mother but was not contacted until palliative nurse went to visit Mum Monday morning and I caught a plane to Auckland.*

Adjusting one’s expectations and understanding in preparation for death was not achievable for some participants in the time available. For example, one participant described how she was told very late in the illness that her mother was dying and shared the regret she felt not being given the opportunity to take her mother home.

*Therefore, when they told me 24 h before she was dying it was a shock. I had some advance warning we may have done things differently. I would have liked to take her home if possible or even discussed this.*

Even when death occurred in a hospice setting, participants often experienced it as sudden and unexpected, despite the progressive nature of the illness. One participant described how they were still making long-term plans despite being aware that their family member was “really sick”. They were kept informed, but not about the possibility of death occurring.

*00083: His care at hospice was without doubt exceptional. Given respect, taken out for smoke breaks whenever he wanted. All family members welcomed and informed, only thing was we didn’t know he would die, were making longer term plans for him! Knew he was really sick but knowing him thought he would outlast us!*

While it can be difficult to predict how someone might respond to medication at the end of life, this uncertainty was not always communicated leading one participant to express regret at not being able to communicate with her mother, which she thought was due to her being given morphine.

*03669: Last 24 h my mother was distressed and wanted to be out of it so nurses gave her medication, morphine. I wish they had told me she may not be able to talk after this as I lost my chance to say goodbye and tell her things. This was a great regret for me. If nurses/dr understood how regret affects someone after their loved one dies surely, they would do it better.*

However, when family were clear about the information they received about care and treatment, the experience was a positive one. One participant described her mother being given opportunities to remain engaged in decisions regarding her care, which was based on mutual respect and trust right up until the end of her life. The sense of certainty that they had done the right thing by their family member, even after death had occurred, was evident in their responses.

*00040: The most striking thing was hearing the doctor explain she was dying, and she could choose how aware she wanted to be. Mum chose Option A., but gave them permission to move to Option B. without further comment from her, trusting then completely to interpret any change from her symptoms. This gave Mum a confident, forward-looking death.*

### Disempowerment and loss of advocacy

Exacerbations related to the illness, and uncertainty regarding prognosis, meant that at times there was uncertainty about the most appropriate place of care. Some participants therefore found it difficult to advocate for their relative and their care. Moving from hospice to home to hospital became a regular event for some people, and some family had a key role in facilitating the most appropriate place of care. “Personal uncertainty” described by Han et al. [16] relates to the psychosocial and existential issues related to the effects of the illness on one’s personal relationships. Increasing concern for others and the impact of the illness on one’s sense of meaning surrounding life in general can result in a sense of “personal uncertainty”. This was evident in the findings when participants described the overwhelming sense of responsibility and advocacy for their family member. One participant described how she had to appeal to a hospital physician to have her mother admitted to hospital:

*00264: She had a week or 10 days in the hospice - then was returned home with a palliative care regime. Finally, the HF episodes became more frequent and she needed hospitalisation but the hospice didn’t have a bed- hence the appeal to the cardiologist and her readmission to hospital. Fantastic that they could ease her last days.*

Some participants expressed uncertainty about whether healthcare professionals had done enough before discharging the patient or moving them to a different place of care. Some participants highlighted that this was even more concerning when their attempts to raise their concerns directly with the doctors and nurses were not heard.

*00600: His care in hospital was excellent to a point, it was when he was in a mainstream ward and not Cardiology that symptoms went unseen. It didn’t seem to make a difference what I was telling staff. There were avenues that should have been investigated that weren’t.*

Some participants also experienced disempowerment in their advocacy role through the manner in which impending death was talked about, which appeared to be as important as when the conversation occurred. One participant described how her mother was told she should not be in hospital as there was no treatment that could be offered, which she found particularly distressing given that her mother was not involved in the conversation.

*02162: So, the young female doctor stood by her bed and said to us there’s no point wasting time and effort on her as she’s going to die. She needs to leave the hospital as we have others who need the bed. She offered no treatment. Mum said “I can hear”. It was the most distressing moment of my life.*

## Discussion

This study explores the impact of uncertainty on bereaved families’ experiences of death and dying in cardiac disease. Details of how stories are told about care at the end of life and how these experiences are remembered provide insight into the ongoing impact of uncertainty on bereaved family. Findings from this study have shown that uncertainty which is not made explicit, can lead to families feeling a pervasive sense of distrust in the information being provided (or not) by healthcare professionals. This contributes to death being experienced as sudden or poorly managed. Over time, as bereaved family try to make sense of and find meaning in their experiences, unanswered questions mean that stories are left incomplete, leading to a sense of missed opportunities, regret, and disempowerment within their advocacy or caring role.

Clinical uncertainty, as reflected in Han et al’s [16] taxonomy of uncertainty in healthcare (Fig. 1), has historically been disease-centered, focusing on the physiological cause of death, as well as prognosis or the timing of death. However, uncertainty related to physiological illness progression may not be a priority for patients and their family, whose concerns Han et al. [16] suggest may be more person-centered. Indeed, findings from this study suggest that priorities, for family of people with heart disease at the end of life, are related to being with and spending time with their ill relative, and being involved in end-of-life planning with an emphasis on the dying process or the kind of dying to expect*.* Furthermore, where the uncertainty of when death might occur is not made explicit, bereaved family are left with memories of loss and regret that are difficult for them to resolve as time goes on.

Findings from this study showed that both the timing and nature of end-of-life conversations between family and healthcare professionals are important to how end of life is experienced, and remembered, by bereaved family. In this study, bereaved family experienced end-of-life conversations as often ill-timed, highlighting that healthcare professionals were often not explicit about uncertainty in terms of death and dying, or the timing of when death may occur. Communicating information regarding the impact of a life limiting illness has received significant attention in the literature [17–19]. However, evidence has shown that conversations with people who have heart failure focus largely on the management of the illness, whilst care at the end of life is rarely discussed [2]. Reasons for this are complex, but healthcare professionals’ reluctance to discuss issues related to death and dying with people with heart failure is suggested to be associated with the unpredictable illness trajectory and prognostic uncertainty [4, 20].

The tension between communicating what may be a lingering or sudden death, both possibilities in people with heart disease, may complicate the way in which impending death is discussed by clinicians, As described above, this study found that the timing of these conversations was particularly important in how family experience death as being sudden or not. Continued misconceptions that actively dying and palliative care are synonymous [21] may contribute to healthcare professionals’ unwillingness to have end-of-life conversations early, when prognosis is uncertain. Indeed, evidence has shown that people with heart disease are far less likely to be enrolled in palliative care services in England [22] or have conversations about end of life with their healthcare professionals [2].

In our study, a focus on disease management combined with a lack of attention to uncertainty regarding illness progression and death was identified as a key finding, shown here to negatively impact bereaved families’ experiences. Furthermore, when conversations with healthcare professionals failed to provide the information family needed, this led to distrust in what was being communicated. A qualitative interview study with surrogate decision-makers by Evans et al. [23] similarly found that trust in healthcare professionals was negatively impacted by lack of discussion about prognostic uncertainty, in highly uncertain end-of-life contexts. Trust is a key aspect not only of relationships between clinicians and patients, but also clinicians and families. However, in general, there is relatively less attention paid in literature to family’s experiences of uncertainty at end of life.

In our study, family particularly highlighted mismatches between the information provided (or not) by healthcare professionals and what family were experiencing; this led to a pervasive sense that healthcare professionals might ‘know something,’ but were not communicating this to patients or family. These findings echo those of a mixed methods study of family’s experiences of hospital end-of-life care by Krawczyk and Gallagher [24]. Although not focused on cardiac disease, they found that when prognostic uncertainty was poorly communicated to family members, family did not understand that their relative was “sick enough to die,” which left a “legacy of uncertainty and suspicion.” As heart failure is most often not treated as terminal, described above, these effects may be further compounded in end of life with cardiac disease.

The mismatches described in our study also created tensions that family had to navigate at the time, and later when making sense of their experiences. For example, when family thought healthcare professionals were not “noticing” the deterioration they themselves could see, family had to decide whether to speak up. Those who spoke up and were not heard felt disempowered; whilst those who chose not to speak up were left with an incomplete narrative characterised by disempowerment and regret. These examples further illustrate how family felt uncertain about what to expect of their relatives’ care, and end of life, impacting how they undertook and remember their caring and advocacy role.

The impact on family when healthcare professionals are not explicit about uncertainty was significant in this study. Feelings of regret and missed opportunity were evident in many participants’ responses. Regret can complicate recovery from bereavement when family do not get the opportunity to be with their relative at the time of death, or do not get a chance to say what they want to say, or do what they had planned to do [25]. Unresolved or unfinished narratives can lead to an inability to make sense of and meaning in their experiences of death and dying, and what might have been.

Communicating uncertainty in the context of a poor prognosis does not align well with the principles of a biomedical model, which are positioned around evidence based practice, science, and certainty. Physicians are trained to find certainty in a diagnosis using best evidence, which is framed around population based studies [26]. Yet, communicating the uncertainty of how the “evidence” might apply to an individual is more complex [27]. Indeed, research has shown that during patient consultations doctors may not communicate clinical uncertainty for fear of negative consequences for the patient, including loss of trust, confusion and anxiety [28].

Alternatively, a focus on preparing for death whenever it may occur (and it will) may help healthcare professionals to understand patient and family wishes, and address their uncertainties in a way that is more person-centered. Indeed, Han and colleagues [16] highlight the importance of acknowledging psychosocial and existential uncertainty, which may be more important to patients and family than scientific uncertainty. A systematic review on end-of-life communication by Parker et al. [17] highlighted that patient and family needs and preferences may diverge, and most family express a wish for candid conversations about prognosis, end of life, and acknowledgement of uncertainty.

Findings from our study have shown that families’ experiences can be profoundly negative if conversations are not conducted well or in a timely manner. Done well, conversations preparing family for impending death can positively shape narratives associated with supporting a family member at the end of life, providing time for them to find meaning and reshape their expectations about the future. Even deaths that are sudden may feel less ‘bewildering’ if conversations are framed around the fact that death will occur at some stage, rather than focusing on exactly when death will occur. Indeed, rather than focusing on prognostication, a better approach may be conversations which are focused on normalizing uncertainty while addressing patients’ and families’ emotions, and supporting them to manage the impact of uncertainty on their day-to-day lives [29].

Considering both healthcare professionals’ concerns and the profound impacts on family, the way in which healthcare professionals manage times of uncertainty are important in achieving a positive outcome for bereaved family. Planning for the worst and hoping for the best means that absolute certainty regarding the timing of death is not necessary – only that they will. Being explicit about the unpredictability of the illness and that healthcare professionals are not able to predict the precise timing of death is an important conversation to have with patients and their family. Being certain about when someone will die is often unattainable; however, certainty of when death may occur may not be as important for some people living with heart failure and their family. There is thus currently a mismatch on the focus and impact of uncertainty for doctors, patients, and family members at the end of life.

Findings from this study have been presented using Han et. al’s [16] conceptual framework of uncertainty in the healthcare context. Uncertainties related to living with a progressive life limiting illness are not all the same, are experienced differently and are neither “stable nor synonymous” [30].

The way in which categories of uncertainty (see Fig. 2) inter-relate reflects the multifaceted and complex nature of uncertainty and its potential to impact on bereaved families experiences of care at the end of life. This study found evidence of participants experiencing the impact of “scientific uncertainty” which was associated with diagnosis, prognosis, casual explanations and treatment. Furthermore, the impact of “practical uncertainty” was revealed when participants expressed distrust in their health care professionals and were left wondering about the quality of care their family member had received. Finally, the category described by Han et al. as “personal uncertainty” relating to psychosocial and existential issues was evident when participants described the overwhelming sense of responsibility they felt and the advocacy they undertook for their family member.

While healthcare professionals continue to focus on when someone is going to die, without acknowledging the uncertainty of prognostication, patients and families will continue to have expectations about death and dying that will remain unresolved. Uncertainty does not negate these conversations if we take practical steps to ensure healthcare professionals understand what is important to patients and family, rather than focusing on trying to make what is uncertain certain.

### Limitations

A key strength of this study is in the collection of data directly from bereaved families who had experienced caring for someone at the end of life with heart disease. However, there are study limitations that should be noted. Firstly, the bereavement period ranged from 6 to 21 months. This may have impacted on participants ability to recall their experiences.

Secondly, it has been argued that the use of survey data from open ended questions may be “uncomfortable to work with” being neither qualitative not quantitative data [31]. However, the use of open-ended questions may provide an opportunity to capture data about an important issue that has not been covered in the survey. This is reflected in the findings from this study whereby the survey did not ask specifically about people’s experiences of uncertainty. Instead, the concept of uncertainty was identified as a key theme during the analysis of open responses.

## Conclusion

This study has highlighted the ongoing impact on bereaved family when uncertainty is not made explicit in conversations regarding end of life for people with heart disease. Timely and sensitive conversations regarding the uncertainty of when death may occur is an important factor in ensuring that bereaved family are not left with unresolved narratives, i.e. feelings of missed opportunities, uncertainty that they had made the right decisions, and lack of clarity around their relative’s death. These impacts may remain present indefinitely. We also offered some reframes for thinking and talking about uncertainty in end of life, as this and other studies have shown that clinicians’ uncertainties may not always reflect or match up with families’ uncertainties. Being explicit about our inability to be certain about the timing of death may thus lead to a more positive and complete experience for bereaved family.

## Data Availability

The datasets used and/or analysed during the current study are available from the corresponding author on reasonable request.
